# Surgical Items

**Published:** 1897-04

**Authors:** 


### Surgical Items.

There is no question that the more cultivated dentists know the surgery of the mouth better than the surgeon who has been only generally trained; know better also the relations of disorders of the oral cavity with continuous and distant tracts, and are better prepared to diagnosticate the cause of many obscure lesions connected with those relations.

I, therefore, recommend to the surgical profession, particularly to those who have had no special opportunities for studying the diseases of the mouth, the calling in of a skillful dentist, preferably one who has been medically educated, at least for the benefit of his judgment in diagnosis, whenever there is room to suspect oral complications.—*Lennox Curtis.*

